# Nine years of mosquito monitoring in Germany, 2011–2019, with an updated inventory of German culicid species

**DOI:** 10.1007/s00436-020-06775-4

**Published:** 2020-07-16

**Authors:** Doreen Werner, Stefan Kowalczyk, Helge Kampen

**Affiliations:** 1 Leibniz Centre for Agricultural Landscape Research, Eberswalder Strasse 84, 15374 Muencheberg, Germany; 2 Friedrich-Loeffler-Institut, Federal Research Institute for Animal Health, Greifswald, Insel Riems Germany

**Keywords:** Culicidae, Germany, Inventory, Mosquito monitoring, Invasive species, Globalisation, Climate warming

## Abstract

Before the background of increasingly frequent outbreaks and cases of mosquito-borne diseases in various European countries, Germany recently realised the necessity of updating decade-old data on the occurrence and spatiotemporal distribution of culicid species. Starting in 2011, a mosquito monitoring programme was therefore launched with adult and immature mosquito stages being collected at numerous sites all over Germany both actively by trapping, netting, aspirating and dipping, and passively by the citizen science project ‘Mueckenatlas’. Until the end of 2019, about 516,000 mosquito specimens were analysed, with 52 (probably 53) species belonging to seven genera found, including several species not reported for decades due to being extremely rare (*Aedes refiki*, *Anopheles algeriensis*, *Culex martinii*) or local (*Culiseta alaskaensis*, *Cs. glaphyroptera*, *Cs. ochroptera*). In addition to 43 (probably 44 including *Cs. subochrea*) out of 46 species previously described for Germany, nine species were collected that had never been documented before. These consisted of five species recently established (*Ae. albopictus*, *Ae. japonicus*, *Ae. koreicus*, *An. petragnani*, *Cs. longiareolata*), three species probably introduced on one single occasion only and not established (*Ae. aegypti*, *Ae. berlandi*, *Ae. pulcritarsis*), and a newly described cryptic species of the *Anopheles maculipennis* complex (*An. daciae*) that had probably always been present but not been differentiated from its siblings. Two species formerly listed for Germany could not be documented (*Ae. cyprius*, *Ae. nigrinus*), while presence is likely for another species (*Cs. subochrea*), which could not be demonstrated in the monitoring programme as it can neither morphologically nor genetically be reliably distinguished from a closely related species (*Cs. annulata*) in the female sex. While *Cs. annulata* males were collected in the present programme, this was not the case with *Cs. subochrea*. In summary, although some species regarded endemic could not be found during the last 9 years, the number of culicid species that must be considered firmly established in Germany has increased to 51 (assuming *Cs. subochrea* and *Ae. nigrinus* are still present) due to several newly emerged ones but also to one species (*Ae. cyprius*) that must be considered extinct after almost a century without documentation. Most likely, introduction and establishment of the new species are a consequence of globalisation and climate warming, as three of them are native to Asia (*Ae. albopictus*, *Ae. japonicus*, *Ae. koreicus*) and three (*Ae. albopictus*, *An. petragnani*, *Cs. longiareolata*) are relatively thermophilic. Another thermophilic species, *Uranotaenia unguiculata*, which had been described for southwestern Germany in 1994 and had since been found only at the very site of its first detection, was recently documented at additional localities in the northeastern part of the country. As several mosquito species found in Germany are serious pests or potential vectors of disease agents and should be kept under permanent observation or even be controlled immediately on emergence, the German mosquito monitoring programme has recently been institutionalised and perpetuated.

## Introduction

Similar to other countries, the list of German culicids was subject to tremendous changes until the second half of the twentieth century regarding scientific names and numbers of species. This was mainly due to taxonomic revisions, including dissolutions of synonymities, restructuring of the generic system and reassignments of species to genera, as well as to descriptions of previously unrecognised, closely related species, including sibling species.

Consequently, Martini (1915), for example, noted 20 mosquito species for Germany, Eckstein (1920) 21, Vogel (1929) 35, Peus (1932) 38, Peus (1937a, 1950) 40, Britz (1955) 42 and Mohrig (1969) 44. In 1968, Peus (1970) added a 45th species. The increase in species numbers is the more impressing as it is in contrast to the reduction in German territory as a consequence of the two world wars (e.g. Eastern Prussia, Eastern Pomerania, Silesia, Alsace-Lorraine). Since all species described for Germany until 1968 had been, and —with the exception of one species (see below)— still are, considered endemic, the preceding recognition of new species was merely a result of scientific progress, i.e. increased knowledge and improved approaches and taxonomic methodologies.

After the disappearance of malaria in the middle of the twentieth century from most of Europe (Bruce-Chwatt and de Zulueta 1980), mosquito research experienced a sharp decline in Germany. Mosquitoes were not considered dangerous vectors anymore and faded from the view of researchers and funders. Data on the mosquito fauna were subsequently collected only locally or, at maximum, regionally, mainly due to being nuisance pests and linked to control activities (e.g. Becker and Ludwig 1983). Thus, Mohrig’s compilation of the ‘Culicidae of Germany’ (Mohrig 1969), which is based both on the literature and the author’s own collections, remained a standard textbook for German mosquito researchers and was referred to as a guide to the biology, occurrence and distribution of German mosquitoes for decades. According to the valid species list of mosquito taxonomic names as of 16 January 2020 (Harbach 2020), it listed 44 species ever reported for Germany and was only supplemented by *Aedes geminus*, described in 1970 as a sister species to *Ae. cinereus* (Peus 1970), and *Uranotaenia unguiculata*, a newly established species detected in 1994 that had invaded from the south (Becker and Kaiser 1995). While additional species were not recognised for almost two decades and those 46 species were listed in a checklist of German dipterans published in 1999 (Dahl et al. 1999), no information was available on the fate of several rare and less common species not encountered for many years.

In 2008, another invasive culicid, *Ae. japonicus*, was found and soon demonstrated to have established (Schaffner et al. 2009; Becker et al. 2011), although already in 2007, eggs of *Ae. albopictus* had been detected in southwestern Germany in an oviposition trap, initially remaining without evidence of establishment (Pluskota et al. 2008).

Triggered by these findings of invasive mosquito species in Germany, which represented potential vectors of disease agents, and the continuing demonstrated spread of *Ae. albopictus* in southern Europe (Scholte and Schaffner 2007; Medlock et al. 2015) as well as increasing numbers of culicid-borne West Nile fever incidents (Hubálek and Halouzka 1999; Bellini et al. 2014) and the emergence of chikungunya and dengue in Europe (Rezza 2014), scientific, political and public interest in mosquitoes experienced a renaissance in Germany and finally resulted in a monitoring programme launched in 2011.

We here present benchmark results of the German monitoring programme, targeting the occurrence and spatiotemporal distribution of both native and invasive species, together with an updated inventory of the mosquito species of Germany. Information on the specific distributions of the species found, including distribution maps and their phenology, will follow separately.

## Materials and methods

### Mosquito collection

Mosquitoes were collected actively and passively. Active collections of adult specimens were carried out by various kinds of traps during the vegetative seasons each year (April to October). At a total of 109 collection sites throughout Germany, BG Sentinel traps (Biogents, Regensburg, Germany) equipped with a CO_2_ source (gas tank releasing 500 g CO_2_/24 h) and baited with BG Lure® (Biogents) as attractants were operated for 24 h per week from April to October during a first monitoring phase (2011–2014). Thirteen of the sites were sampled for 4 years, 41 sites for 3 years, 15 sites for 2 years and 40 sites for 1 year. In addition to the BG Sentinels, CO_2_-baited EVS (encephalitis virus surveillance) traps (BioQuip Products, Compton, CA, USA) (with the equivalent concentration of released gas) were used in the same rhythm (i.e. once per week for 24 h from April to October) at 52 sites, with 15 sites sampled for 2 years and 37 sites sampled for 1 year. In a second project phase (2015–2017), 64 BG Sentinels were annually operated for 24 h once per month in April and October and twice per month from May to September at different sites per year in the eastern half of Germany. For organisational reasons, no systematic trapping took place in 2018, but ten EVS traps each were operated for 2 to 3 weeks on four limited areas in eastern Germany following West Nile virus (WNV) emergence (Kampen et al. 2020). In 2019, systematic trapping covering the complete vegetative season was resumed with 31 BG Sentinels, run at more or less evenly distributed sites throughout Germany which promised to offer a high species diversity and a high abundance of mosquitoes, and an additional 20 EVS traps at a WNV hotspot in the Wildlife Park Berlin in September (Kampen et al. 2020).

Furthermore, adult mosquitoes were caught throughout Germany by aspirating females from resting places in animal (predominantly sheep, goat, cattle and horse) shelters and zoo settings (305 collections, 58 stables, barns, etc.) (Heym et al. 2018; Werner and Kampen, unpublished) and hibernating females in winter shelters, such as caves, dungeons and cellars (137 objects, with one object sampled twice), as well as by netting from bushy vegetation (e.g. in forests) and from both animal and human baits (Werner and Kampen, unpublished).

Finally, mosquito larvae and pupae were actively collected throughout Germany by dipping and sieving in natural and artificial breeding places (ca. 3000 collections at 1500 sites).

To enlarge the data pool and support data collection in terms of quantity (number of mosquitoes) and quality (species spectrum), mosquitoes were also collected passively by a citizen science project, the ‘Mueckenatlas’, which had been launched in April 2012 (Kampen et al. 2015; Walther and Kampen 2017). In that project, citizens are asked to capture mosquitoes in their private surroundings and submit them for scientific analysis.

### Mosquito identification

For practical reasons, morphological determination of mosquitoes was generally performed on adult specimens using the identification keys by Mohrig (1969), Schaffner et al. (2001) and Becker et al. (2010). Collected immature stages were therefore kept in jars and beakers containing water taken from their breeding sites until adult emergence. Adult mosquito specimens were killed by freezing for at least 1 h at − 20 °C.

Individuals belonging to the *Culex pipiens* and *Anopheles maculipennis* complexes were genetically identified by species-specific PCR assays (Proft et al. 1999; Rudolf et al. 2013; Kronefeld et al. 2014). Other closely related species or damaged specimens that could not be identified based on morphological characters were subjected to CO1 (cytochrome *c* oxidase subunit 1) PCR and sequencing (Folmer et al. 1994; Hébert et al. 2003).

### Data management

All collection data were fed into the German mosquito database CULBASE which, for the purpose of the present contribution, was filtered for mosquito species and developmental stage, number of collection sites and mode of sampling.

## Results and discussion

Within the scope of the present monitoring project, a total of more than 516,000 mosquito specimens collected from 2011 to 2019 throughout Germany were analysed, including some 300,000 trapped specimens, ca. 62,000 adult hand catches (aspirated, netted), ca. 137,000 mosquitoes submitted to the ‘Mueckenatlas’ scheme and at least 17,000 immature stages collected from their breeding sites. The number of collected immature stages was actually much higher than 17,000 as often only one larva per species, site and collection event was entered into the database, although more specimens had been collected and identified. This discrepancy is due to non-standardised collection efforts and time.

**Fig. 1:**
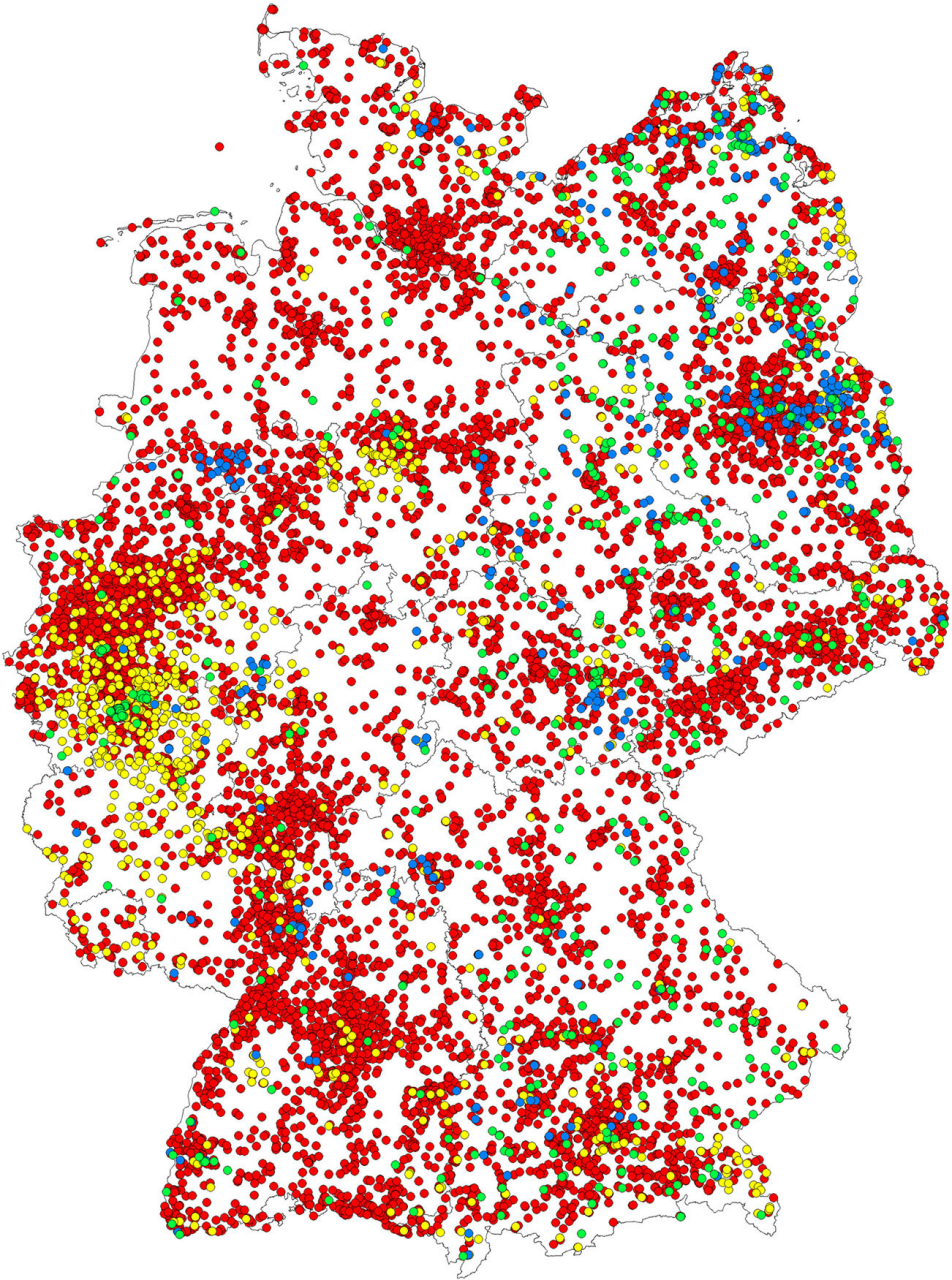
Mosquito collections 2011–2019 (green dots—trap collections; red dots—‘Mueckenatlas’ submissions; blue dots—netting, aspirating and baited collections of adults; yellow dots—dipping/sieving of immature developmental stages)

The mosquitoes belonged to an assured 52 species out of seven culicid genera and were collected at roughly 22,600 sites (Table 1, Fig. 1). Probably, specimens of a 53rd species (*Cs. subochrea*) were among the collections, but this species could not be reliably distinguished from a closely related species (*Cs. annulata*) in the life stages available. Forty-three (possibly 44, assuming the presence of *Cs. subochrea*) of the collected species were included in the 46 thought to occur in Germany prior to the onset of the monitoring programme (Dahl et al. 1999). In addition, several invasive species were captured, five of which are now considered established: *Culiseta longiareolata*, *An. petragnani*, *Ae. albopictus*, *Ae. japonicus* and *Ae. koreicus.*

**Table 1:** Mosquito species listed for Germany by Dahl et al. (1999) and found within the scope of the presented monitoring programme (non-native species with single detections excluded)

Species	First documented on the territory of present-day Germany^1^	Found last in Germany (documented last^2^)	Found in this monitoring project			Considered established
			Adults		Immature stages	
			Trap	Mueckenatlas		
*Ae.* (*Stegomyia*) *albopictus* Skuse, 1895	2007 (Pluskota et al. 2008) (as *Stegomyia albopicta*)	2019 (this study)	+	+	+	+
*Ae.* (*Ochlerotatus*) *annulipes* Meigen, 1830	1898 (Mohrig 2000) (as *Cx. annulipes*)	2019 (this study)	+	+	+	+
*Ae.* (*Ochlerotatus*) *cantans* Meigen, 1818	1906 (Eysell 1907) (as *Cx. cantans*)	2019 (this study)	+	+	+	+
*Ae.* (*Ochlerotatus*) *caspius* Pallas, 1771	1928 (Peus 1929)	2019 (this study)	+	+	+	+
*Ae.* (*Ochlerotatus*) *cataphylla* Dyar, 1916	1920 (Martini 1920b) (as *Ae. rostochiensis*)	2019 (this study)	+	+	+	+
*Ae.* (*Aedes*) *cinereus* Meigen, 1818	1897 (Mohrig 2000)	2019 (this study)	+	+	+	+
*Ae.* (*Ochlerotatus*) *communis* de Geer, 1776	1896 (Mohrig 2000)	2018 (this study)	+	+	+	+
*Ae.* (*Ochlerotatus*) *cyprius* Ludlow, 1920	1900 (Edwards 1921) (as *Ae. freyi*)	1925 (Peus 1937b)	−	−	−	−^3^
*Ae.* (*Ochlerotatus*) *detritus* Haliday, 1833	1919 (Martini 1920b) (as *Ae. salinus*)	2019 (this study)	+	+	+	+
*Ae.* (*Ochlerotatus*) *diantaeus* Howard, Dyar & Knab, 1913	1893 (Mohrig 2000)	2017 (this study)	+	+	−	+
*Ae.* (*Ochlerotatus*) *dorsalis* Meigen, 1830	1914 (Martini 1920b)	2019 (this study)	+	+	+	+
*Ae.* (*Ochlerotatus*) *excrucians* Walker, 1856	1898 (Mohrig 2000)	2019 (this study)	+	+	+	+
*Ae.* (*Ochlerotatus*) *flavescens* Müller, 1764	1895 (Mohrig 2000)	2019 (this study)	+	+	+	+
*Ae.* (*Aedes*) *geminus* Peus 1970	1968 (Peus 1970)	2019 (this study)	+	+	+	+
*Ae.* (*Dahliana*) *geniculatus* Olivier, 1791	1898 (Mohrig 2000) (as *Cx. ornatus*)	2019 (this study)	+	+	+	+
*Ae.* (*Ochlerotatus*) *intrudens* Dyar, 1919	1928 (Peus 1929)	2018 (this study)	+	+	+	+
*Ae.* (*Hulecyteomyia*) *japonicus* Theobald, 1901	2008 (Schaffner et al. 2009)	2019 (Kampen et al. 2020)	+	+	+	+
*Ae. (Hulecyteomyia*) *koreicus* Edwards, 1917	2015 (Werner et al. 2016)	2019 (this study)	−	+	+	+
*Ae.* (*Ochlerotatus*) *leucomelas* Meigen, 1804	1896 (Mohrig 2000)	2019 (this study)	+	+	+	+
*Ae.* (*Ochlerotatus*) *nigrinus* Eckstein, 1918	1932 (Peus 1933)	1993 (Becker and Kaiser 1995)	−	−	−	?
*Ae.* (*Ochlerotatus*) *pullatus* Coquillett, 1904	1916 (Kühlhorn 1954)	2016 (this study)	+	+	+	+
*Ae.* (*Ochlerotatus*) *punctor* Kirby, 1837	1913 (Kühlhorn 1954)	2019 (this study)	+	+	+	+
*Ae.* (*Rusticoidus*) *refiki* Medschid, 1928	1897 (Vogel 1931)	2017 (Kuhlisch et al. 2017)	+	+	+	+
*Ae.* (*Ochlerotatus*) *riparius* Dyar & Knab, 1907	1919 (Martini 1920a) (as *Ae. semicantans*)	2019 (this study)	+	+	+	+
*Ae.* (*Aedes*) *rossicus* Dolbeskin, Gorickaja & Mitrofanova, 1930	1963 (Müller 1965)	2019 (this study)	+	+	+	+
*Ae.* (*Ochlerotatus*) *rusticus* Rossi, 1790	1914 (Martini 1920a) (as *Ae. diversus*)	2019 (this study)	+	+	+	+
*Ae.* (*Ochlerotatus*) *sticticus* Meigen, 1838	1901 (Mohrig 2000) (as *Cx. sticticus*)	2019 (this study)	+	+	+	+
*Ae.* (*Aedimorphus*) *vexans* Meigen, 1830	1900 (Mohrig 2000) (as *Cx. vexans*)	2019 (this study)	+	+	+	+
*An.* (*Anopheles*) *algeriensis* Theobald, 1903	1931 (Martini 1931)	2019 (this study)	+	+	−	+
*An.* (*Anopheles*) *atroparvus* van Thiel, 1927	1931 (Martini et al. 1931)	2016 (Kampen et al. 2016b)	+	+	+	+
*An.* (*Anopheles*) *claviger* Meigen, 1804	1911/12 (Schneider 1913) (as *An. bifurcatus*)	2019 (this study)	+	+	+	+
*An.* (*Anopheles*) *daciae* Linton, Nicolescu & Harbach, 2004	2007 (Weitzel et al. 2012)	2019 (this study)	+	+	+	+
*An.* (*Anopheles*) *maculipennis* Meigen, 1818	1931 (Martini et al. 1931)	2019 (this study)	+	+	+	+
*An.* (*Anopheles*) *messeae* Falleroni, 1926	1931 (Martini et al. 1931)	2019 (this study)	+	+	+	+
*An.* (*Anopheles*) *petragnani* del Vecchio, 1939	2015 (Becker et al. 2016, Kampen et al. 2017)	2019 (this study)	+	+	+	+
*An.* (*Anopheles*) *plumbeus* Stephens, 1828	1911 (Schneider 1913) (as *An. nigripes*)	2019 (this study)	+	+	+	+
*Cq.* (*Coquillettidia*) *richiardii* Ficalbi, 1889	1896 (Mohrig 2000)	2019 (this study)	+	+	+	+
*Cx.* (*Culex*) *hortensis* Ficalbi, 1889	1927 (Peus 1929)	2019 (this study)	+	+	+	+
*Cx.* (*Culex*) *martinii* Medschid, 1930	1935 (Peus 1950)	2017 (Kuhlisch et al. 2018b)	+	−	−	+
*Cx.* (*Culex*) *modestus* Ficalbi, 1890	1960/61 (Mohrig 1963)	2019 (Kampen et al. 2020)	+	+	+	+
*Cx.* (*Culex*) *pipiens* Linnaeus, 1758	1900 (Mohrig 2000)	2019 (Kampen et al. 2020)	+	+	+	+
*Cx.* (*Culex*) *territans* Walker, 1856	1911 (Schneider 1913)	2019 (this study)	+	+	+	+
*Cx.* (*Culex*) *torrentium* Martini, 1925	1924 (Martini 1924b)	2019 (Kampen et al. 2020)	+	+	+	+
*Cs.* (*Culiseta*) *alaskaensis* Ludlow, 1906	1897 (Mohrig 2000) (as *Cx. annulatus*)	2017 (this study)	+	+	+	+
*Cs.* (*Culiseta*) *annulata* Schrank, 1776	1896 (Mohrig 2000) (as *Cx. annulatus*)	2019 (this study)	+	+	+	+
*Cs.* (*Culicella*) *fumipennis* Stephens, 1825	1930 (Peus 1950)	2019 (this study)	+	?	?	+
*Cs.* (*Culiseta*) *glaphyroptera* Schiner, 1864	1923 (Martini 1924b)	2018 (this study)	+	+	+	+
*Cs.* (*Allotheobaldia*) *longiareolata* Macquart, 1838	2011 (Becker and Hoffmann 2011, Werner et al. 2012)	2019 (this study)	+	+	+	+
*Cs.* (*Culicella*) *morsitans* Theobald, 1901	1911 (Schneider 1913) (as *Culicada morsitans*)	2019 (this study)	+	+	+	+
*Cs.* (*Culicella*) *ochroptera* Peus 1935	1928 (Peus 1935) (as *Theobaldia. glaphyroptera*)	2017 (Kuhlisch et al. 2019)	+	+	+	+
*Cs.* (*Culiseta*) *subochrea* Edwards 1921	1914 (Martini 1924a) (as *Th. annulata* var. *ferruginata*)	1991 (Becker and Kaiser 1995)	?	?	?	+
*Ur.* (*Pseudoficalbia*) *unguiculata* Edwards, 1913	1994 (Becker and Kaiser 1995)	2019 (this study)	+	−	+	+
Total number of species	52		48 (49?)	46 (48?)	45 (47?)	50
			49 (50?)			

+/–: included/not included in the collections; ?: reliable identification not possible due to high morphological similarity and CO1-DNA sequence homology with other species, lack of males and processing of adults only; ^1^based on literature published from the year 1900 onwards; ^2^references provided only when most recent documentation occurred before most recent finding in the present monitoring programme; ^3^should not be considered belonging to the German mosquito fauna anymore

*Culiseta longiareolata*, a thermophilic species widely distributed in the Mediterranean (Becker et al. 2010), was discovered in southwestern Germany in 2011 (Becker and Hoffmann 2011; Werner et al. 2012; Kampen et al. 2013b). In the following years, it was repeatedly found in more central and northern parts of Germany (Kampen et al. 2017), while in 2018 and 2019, it was for the first time demonstrated at exactly the same places in the West German cities of Worms and Alzey (cemeteries) as in 2017, indicating overwintering and establishment (Kampen and Werner, unpublished data).

*Anopheles petragnani* is another thermophilic species which predominantly occurs in southwestern Europe (Becker et al. 2010). In Germany, it was first detected in 2015 (Becker et al. 2016) but has since been reported from four sites in the southern half of the country, with annual larval collections at one of these sites (rock pools in the river Murg close to the city of Forbach in the federal state of Baden-Württemberg) from 2015 to 2019, indicating persistent maintenance (Kampen et al. 2017; Kampen and Werner, unpublished data).

The Asian tiger mosquito *Ae. albopictus*, the most invasive mosquito species of the world within the last 30 years or so (Paupy et al. 2009), has been reported from 28 European countries in 20 of which it succeeded in establishing (Medlock et al. 2015; Robert et al. 2019). By producing diapausing eggs, this thermophilic species has become adapted to more temperate climates, and a strong tendency to spread northwards can be observed (e.g. Armstrong et al. 2017). *Aedes albopictus* was initially trapped in the southwestern part of Germany only, predominantly on service stations along motorways entering the country from the south (Werner et al. 2012; Becker et al. 2013; Kampen et al. 2013a). These findings were generally attributed to introductions by vehicle transport from southern Europe, such as Italy, where the species is widely distributed (Romi et al. 2008). More recently, *Ae. albopictus* was increasingly often reported from other parts of southern Germany and remote from motorways, linked to local reproduction over extended periods of time (Werner and Kampen 2015). Repeated overwintering suggests establishment of the species at various localities in Germany, including the northernmost population worldwide (Pluskota et al. 2016; Becker et al. 2017; Walther et al. 2017; Kuhlisch et al. 2018a).

A fourth invasive and established species found, *Ae. japonicus*, originates from East Asia and is well adapted to temperate climates (Kampen and Werner 2014; Kaufman and Fonseca 2014). The first records within the present monitoring programme were again from southwestern Germany (Werner et al. 2012) where the species had been known to occur since 2009 (Schaffner et al. 2009; Becker et al. 2011). In 2012, 2013 and 2015, specimens collected in western, northern and southeastern Germany were submitted to the ‘Mueckenatlas’, resulting in the detection of three additional, previously unknown populations of the species (Kampen et al. 2012; Werner and Kampen 2013; Zielke et al. 2016). The southwest German population of *Ae. japonicus* included, which spread to France in the west and to Switzerland in the south, four populations of *Ae. japonicus* existed, completely or partly, on German territory in 2015 (Kampen and Werner 2014; Zielke et al. 2016). Both ‘Mueckenatlas’ submissions and field collections from 2016 from outside the known population areas suggested that the species kept spreading (Kampen et al. 2017). As of 2017, the various German populations had either merged or were close to merging, with numerous collection sites throughout the southern half of Germany, although much more dense in the western part (Koban et al. 2019). This process continued until 2019 when putative gaps in the distribution map of *Ae. japonicus* in southern Germany filled (Kampen et al. 2020).

Finally, *Ae. koreicus* succeeded in establishing in Germany. After the first finding of a specimen in Bavaria in 2015 (Werner et al. 2016), the species emerged in the federal state of Hesse in 2016 where it could be found again in 2017 and 2018 at several places, apparently having built up a population (Pfitzner et al. 2018; Steinbrink et al. 2019).

In addition to the invasive thermophilic species, climate warming obviously also has an impact on thermophilic mosquito species already present in Germany, such as *Ur. unguiculata*, which is widely distributed in the Mediterranean (Becker et al. 2010). This species was first detected in Germany in 1994 in the northern Upper Rhine valley (Becker and Kaiser 1995) and from then on repeatedly encountered at that very same site, but nowhere else. Only in 2016 (two sites), and again in 2017, 2018 and 2019 (one site each), the species was discovered, both as larvae and as adults, in northeastern Germany (Tippelt et al. 2017; Werner and Kampen, unpublished data).

Single specimens of three further non-indigenous species, *Ae. aegypti*, *Ae. berlandi* and *Ae. pulcritarsis*, were demonstrated on one occasion each in 2016 after submission to the ‘Mueckenatlas’ scheme (Kampen et al. 2016a, 2017; Werner and Kampen, unpublished) and can therefore not be considered belonging to the German mosquito fauna.

However, an eighth species previously not listed although probably present was demonstrated to occur (Kronefeld et al. 2012, 2014): *Anopheles daciae*, a cryptic species of the *An. maculipennis* complex which was only separated from its sibling, *An. messeae*, in 2004 on the basis of fixed genetic differences (Nicolescu et al. 2004). The species does not appear to be particularly rare in Germany, but seems to have major distribution areas in southern and northeastern Germany (Kronefeld et al. 2014; Kampen et al. 2016b; Lühken et al. 2016; Czajka et al. 2020; Kampen and Werner, unpublished data). Findings from outside Germany suggest that *An. daciae* is much more frequent and widespread in Europe than initially assumed (e.g. Rydzanicz et al. 2017; Blažejová et al. 2018>; Kavran et al. 2018; Culverwell et al. 2020). Coincidently with the detections of *An. daciae* in Germany, documentations of *An. atroparvus,* another member of the *An. maculipennis* complex, have become quite rare.).

Among the 52 (53 including *Cs. subochrea*) species found, several less frequent and even rare species were registered (Kampen et al. 2014), including three *Culiseta* species which had not been reported for decades: *Cs. alaskaensis*, *Cs. glaphyroptera* and *Cs. ochroptera* (Kampen et al. 2013b; Kuhlisch et al. 2019). Extremely rare species re-discovered are *Ae. refiki*, *An. algeriensis* and *Cx. martinii* (Krüger and Tannich 2014; Kuhlisch et al. 2017, 2018b; Tippelt et al. 2018)*.*

Two species listed in previous mosquito checklists were not found at all within the scope of the monitoring project: *Ae. cyprius* and *Ae. nigrinus*. In the case of *Ae. cyprius*, it is highly questionable whether it still occurs in Germany. It had been found only in the mid-1920s at very few sites around Berlin (Peus 1937b), and not much later, Peus (1950) already considered it extinct. *Aedes nigrinus* is another extremely rare species, which—to the best of the authors’ knowledge—was documented four times only for Germany, each time with larval findings: twice in the 1930s from northwestern Baden-Württemberg and Upper Bavaria (Peus 1933, 1950), once in 1980 from Central Germany (Heitkamp et al. 1985), although the species identification in that case must be doubted as the biotopes (forest ponds) were untypical, and once and last in 1993 with one single specimen found in the Upper Rhine valley (Becker and Kaiser 1995).

It is unclear whether *Cs. subochrea* was or was not found during the presented monitoring programme due to close relatedness and high morphological and genetic similarity to *Cs. annulata*. While several *Cs. annulata* males could be unambiguously identified based on characteristics of their genitalia, this was never the case with *Cs. subochrea*. According to the original literature on German mosquitoes, *Cs. subochrea* was observed last in 1991 (Becker and Kaiser 1995). However, although the latter species apparently is not very common, it is supposed to be widely distributed in Europe and can be assumed to still occur in Germany.

While 48 (probably 49 including *Cs. subochrea*) of the registered species had been trapped, 49 species (51 including *Cs. subochrea* and *Cs. fumipennis*) had been collected and submitted by citizens to the ‘Mueckenatlas’ (Table 1). *Culex martinii* was the only species only caught by traps (disregarding *Cs. fumipennis* and *Cs. subochrea* of which no pertinent data exist) while *Ae. aegypti* and *Ae. koreicus* adults were only obtained via the ‘Mueckenatlas’, but not by trapping. Although both active and passive approaches collected almost the same number of species until the end of 2019, the detection of new introductions or populations of invasive *Aedes* species could almost always be credited to the ‘Mueckenatlas’ scheme (Werner and Kampen, unpublished).

As opposed to all established species caught as adults, 45 of them (47 including *Cs. subochrea* and *Cs. fumipennis*) were collected as aquatic stages (i.e. larvae and pupae). *Aedes detritus*, *Ae. diantaeus* and *An. algeriensis*, which were collected as adults, were not represented among the immature mosquito stages.

Not surprisingly, females of *Cx. pipiens* s.l., *An. maculipennis* s.l., *Anopheles* sp., *Aedes* sp. and *Culiseta* sp. were found in winter shelters, but their analysis according to species level remains to be done.

Species identification was difficult or impossible in some groups of closely related species, when morphological characters were missing or ambiguous. Thus, *Ae. annulipes*/*cantans*/*excrucians*/*riparius*, *Ae. cataphylla*/*leucomelas*, *Ae. cinereus*/*geminus*/*rossicus*, *Ae. intrudens*/*diantaeus*, *Cs. morsitans*/*fumipennis* and, as already mentioned, *Cs. annulata*/*subochrea* could often not be differentiated, even by CO1-barcoding. For these groups of species, it would be rather helpful to have reliable species-specific genetic markers at hand.

## Conclusion

The German mosquito monitoring programme provided valuable data as to the present occurrence of culicid species in Germany. Apparently, changes in the mosquito fauna have recently occurred, not least caused by the introduction, establishment and spread of invasive species. These changes therefore mainly apply to additional species rather than lost species. It is evident that environmental and ecological changes have an impact on the availability of habitats for some specialised and stenoecious mosquito species (e.g. *An. atroparvus*, *Cs. ochroptera* and *Cs. glaphyroptera*, which have become rare in Germany due to a loss of suitable breeding sites), but these do not seem to have resulted in species extinction yet. The two species listed in previous checklists but neither found during this monitoring programme nor documented by others in Germany for decades, *Ae. cyprius* and *Ae. nigrinus*, are breeders of pools in open landscapes such as meadows and floodplains, which are still existent. Thus, their absence, or lack of finding, may have other reasons. In summary, the current number of culicid species established in Germany amounts to 51 (Table 1).

As *Ae. albopictus*, *Ae. japonicus* and *Ae. koreicus* are spreading and, together with various indigenous taxa (e.g. *Ae. vexans*, *An. maculipennis* s.l., *Cx. modestus*, *Cx. pipiens* s.l.), may pose a risk to human and animal health (Kampen and Walther 2018), the German mosquito monitoring programme has recently been institutionalised and perpetuated with the aim of collecting, assessing and distributing data on spatial occurrence, seasonal population dynamics and abundance of both native and invasive species.
